# High Emotional Demands at Work and Poor Mental Health in Client-Facing Workers

**DOI:** 10.3390/ijerph19127530

**Published:** 2022-06-20

**Authors:** Chunhui Suh, Laura Punnett

**Affiliations:** 1 Department of Occupational and Environmental Medicine, Institute of Environmental and Occupational Medicine, Pusan Paik Hospital, Inje University, 75 Bokji-ro, Busanjin-gu, Busan 47392, Korea; 2 Department of Biomedical Engineering, University of Massachusetts Lowell, Lowell, MA 01854, USA; laura_punnett@uml.edu; 3 Center for the Promotion of Health in the New England Workplace (CPH-NEW), University of Massachusetts Lowell, Lowell, MA 01854, USA

**Keywords:** anxiety, customer service, depression, emotional demand, emotional labor, mental health

## Abstract

This study investigated the association between emotional demands and depression or anxiety in a wide range of jobs. We used data from the third Korean Working Conditions Survey (*n* = 50,032) for all occupational classifications, with no limitations placed on job title or employment type. Among the full set of regular paid workers in addition to self-employed, unpaid family workers, and informal employees such as independent contractors, 23,989 respondents worked with “customers, passengers, students, or patients” (i.e., clients). Emotional demands were evaluated using two questions: handling angry clients and needing to hide feelings for work performance. Any depression or anxiety over the last 12 months was taken to indicate poor mental health. Multivariable logistic regression modeling was performed to calculate adjusted ORs with 95% confidence intervals for the influence of emotional demands on mental health, adjusting for demographic factors (age, gender, education, income), occupational psychological demands, decision latitude, social support, weekly work hours and job insecurity. The prevalence of emotional demands was higher in self-employed and informal employees than in regular paid employees. The more frequent the exposure to the two emotional demands combined was, the higher the risk of depression or anxiety. High psychological demands, low social support, and low job security each further increased the risk of poor mental health. Emotional demands turned out to be widespread in the entire economy, were not limited to service or sales occupations, and were more evident in precarious work. The contribution of emotional demands and other preventable job stressors to the burden of depression or anxiety in society may be substantial.

## 1. Introduction

With rising employment in the service sector [1], there has been increased attention given to emotional work demands, or emotional labor, and related health issues that result from interactions between frontline service workers and clients. The sixth European Work Conditions Survey (EWCS) report [2] defined emotional demands as having three components, all of which require efforts to manage emotions: (1) hiding your feelings at work; (2) handling angry clients, customers, patients, pupils, etc.; and (3) being in situations that are emotionally disturbing.

Having to handle angry clients is an occupational psychosocial hazard which has reportedly become more common since 2010 [2]. In this process, hiding one’s feelings is an emotional regulation process [3] that affects a range of outcomes such as burnout [4], depressive mood [5], and mental health [6,7]. Two previous Korean studies have reported the combined effects of handling angry clients and hiding one’s feelings of depression or anxiety, with some moderation by other job features [8,9]. The detrimental effects of emotional demands on employee well-being [10] negatively affected work engagement, including vitality at work [11]. In a Danish study, the combination of emotionally disturbing situations, emotionally demanding work and emotional involvement in work was associated with a 1.5-fold increase in risk of long-term sickness absence [12]; this combination was also associated with a 1.19 or 1.32-fold increase in risk of hospital-treated depressive disorder [7].

Most research on emotional demands and mental health has been conducted within specific occupations such as healthcare workers [13,14,15] and call center workers [16,17]. Even studies using national databases have typically been limited to subjects within the occupation categories of sales and service [9,18]. However, many workers outside those standard classifications also interact with customers, patients, pupils, and other clients. Thus, past studies may have overlooked relevant jobs and underestimated the prevalence of affected workers. For example, according to the EWCS [2], the proportion of workers who hide emotions or handle angry clients was high for managers, professionals and technicians—a group that includes teachers and healthcare managers. Furthermore, past studies typically included only paid employees and excluded self-employed, unpaid family workers and informal employees such as independent contractors, even though they also interact with clients and have shown an increased risk of depression, anxiety and sleep disorders [6,8,9,12,19].

In this study, we sought to cover the wide range of occupations that might involve dealing with clients, regardless of specific occupational classification or employment status. We also located emotional demands within the context of a comprehensive assessment of psychosocial working conditions. The aim was to investigate the separate, combined and interacting effects of hiding feelings and handling angry clients on poor mental health, considering other modifiable psychosocial factors at work.

## 2. Materials and Methods

### 2.1. Data Collection

This study analyzed data from the third Korean Working Condition Survey (KWCS) conducted in 2011 by the Korea Occupational Safety and Health Agency. The survey population was a nationally representative sample of the entire working population in all households residing in South Korea, as of the time of the survey. The study design involved multistage random sampling and used the Enumeration Districts developed for sampling in the 2005 Population and Housing Census. Eligible individuals had worked for at least one hour, with some form of compensation, in the week preceding the survey. Professional surveyors conducted face-to-face interviews in 16 major Korean cities/provinces in June–October 2011. A detailed description of the third KWCS was published in the Working Conditions Survey Report [20]. The validity and reliability of the KWCS was reported in a previous study [21]. The protocol of this study was reviewed and approved by the Institutional Review Board of Inje University Busan Paik Hospital (IRB File No. BPIRB 2021-12-080).

### 2.2. Subjects

**Figure 1:**
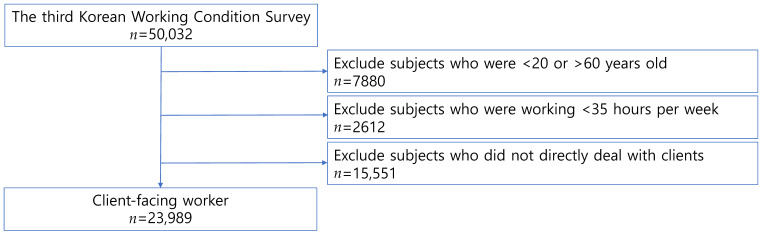
Study subjects.

In the present study, from the 50,032 individuals enrolled in the third wave of the KWCS, we obtained data for those aged 20 to 60 years old and working at least 35 h weekly. To identify potentially exposed workers, we used the statement: “I directly manage those who are not business partners, such as customers, passengers, students, or patients”. (Henceforth, we refer to these four groups together as “clients”). The seven response options were: always, almost always, 75% of the time, 50% of the time, 25% of the time, almost never, and never. We excluded workers who managed clients “almost never” or “never”. In total, 48% of all respondents (*n* = 23,989) were included in the study (Figure 1). Of note, 53% of those who reported attending to clients in their jobs were in occupational categories outside the “sales” and “service” classifications.

### 2.3. Measurements

#### 2.3.1. Outcome Variables

Depression and anxiety were evaluated using the question “Over the last 12 months, did you suffer from any depression or anxiety?” Those who answered “yes” were combined into a group with poor mental health.

#### 2.3.2. Exposure Variables

The level of emotional demands was assessed through the combination of handling angry clients (HAC) and hiding feelings (HF). HAC was measured by the question: “Does your main job involve handling angry clients, patients?” The seven answer categories were the same as for frequency of handling any clients (see above). We categorized HAC into 50% of the time or higher, 25% of the time, and almost never or never. HF was measured from the statement “Your job requires that you hide your feelings” with five response categories: (1) always, (2) most of the time, (3) sometimes, (4) rarely, and (5) never. This job feature is often referred to as surface-acting emotional labor [11]. We categorized HF into “high” (1 and 2) and “low” (3–5). A six-level interaction term was defined using all possible combinations of HAC and HF.

Demographic variables of gender, age, education, and income were considered potential confounding factors. Study subjects were divided into four age groups: 20–29, 30–39, 40–49, and 50–60 years; four educational levels: middle school or below, high school, 2 years in college, and 4 years in university or more; and four monthly income levels: KRW < 1.3 million, KRW 1.3 to less than 2.0 million, KRW 2.0 to less than 3.0 million, and KRW ≥ 3.0 million. General health status was categorized into three groups: (1) good; (2) fair; and (3) bad.

Employment status was categorized into five groups: (1) self-employed without employees; (2) self-employed with employees; (3) employees; (4) unpaid family workers; (5) other employees not included in any previous category, e.g., independent contractor (such as a freight truck driver or quick servicer). Occupation was categorized into ten groups according to the sixth Korean Standard Classification of Occupation: (1) managers, (2) professionals and related workers, (3) clerks, (4) service workers, (5) sales workers, (6) skilled agricultural, forestry and fishery workers, (7) craft and related trade workers, (8) equipment, machine operating and assembling workers, (9) elementary workers, and (10) armed forces.

Psychosocial factors at work were assessed by psychological demands (four items) and decision latitude (three items for skill discretion and six items for decision authority), social support from colleagues and supervisors (one item each), weekly working hours (35–40, 41–48, 49–54, 55–60, 61 and over) and job insecurity (one item, to lose one’s job in the next 6 months). The items from the third KWCS questionnaire that were used to construct these psychosocial factors are presented in the Appendix A. Job strain was determined by the combination of psychological demands and decision latitude (low-strain group = low psychological demand + high decision latitude; active group = high psychological demand + high decision latitude; passive group = low psychological demand + low decision latitude; high-strain group = high psychological demand + low decision latitude) [22]. As the number of response categories varied from one item to another, Niedhammer’s formula [23] was used to standardize all the scores, calculating a new value = 1 + (value − 1)/(the number of response categories of given item − 1). Each new psychosocial score ranged from 1 to 2.

### 2.4. Statistical Analysis

**Figure 2:**
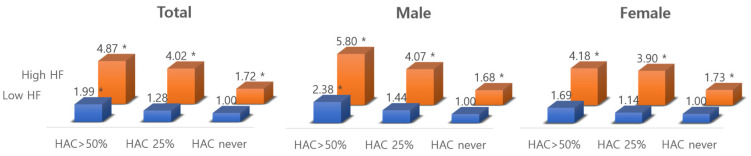
The interactive effects of handling angry clients x hiding feelings on mental health, stratified by gender. * *p*-value below 0.05 for adjusted odds ratio.

**Figure 3:**
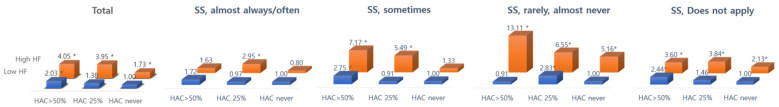
The interactive effects of handling angry clients x hiding feelings on mental health, stratified by social support from supervisors. * *p*-value below 0.05 for adjusted odds ratio.

Descriptive statistics and bivariate analyses were reviewed to assess range and power for analysis. Among the exposure variables, crude predictors of poor mental health (chi-squared test, *p*-values ≤ 0.10) were retained in the multivariate model to ensure that their effects were taken into account. Multivariable logistic regression modeling was used to calculate adjusted ORs with 95% confidence intervals (*CI*s) of emotional demands on mental health, while adjusting for age, gender, education, income, general health status and other psychosocial factors at work. We analyzed the interactive effects of handling angry clients and hiding feelings on mental health stratified by gender (Figure 2) and social support from supervisor (Figure 3). The *p*-values for the interactive effects were estimated through multivariable logistic regression models and adjusted for the same variables as above. All statistical analyses were undertaken using SPSS version 26.

## 3. Results

In the total study population of client-facing workers (*n* = 23,989), 53% were male and the average age of participants was 42 years old (Table 1). About half had at least some college education. Respondents were spread evenly across levels of income. Over 70% of respondents had a good general health status. Paid employees accounted for 57% of the total; service and sales workers were 47%. Half of respondents had high psychological demands and 55% had low decision latitude. High social support from colleagues was more common (42%) than that from supervisors (30%). About 45% worked for 55 h or more per week, and about 3% expected to lose their job in the next 6 months.

About 11% of all workers handled angry clients during more than half of their work time; this was more common among paid employees and among those classified as clerks, service workers, and equipment operators (Table 2). Hiding feelings always or most of the time was reported by 34% overall, especially among the self-employed and informal or other employees, professionals, service and sales workers. Overall, more than half of those reporting either of these emotional demands were in occupational classifications other than service or sales.

A total of 334 (1.4%) client-facing individuals experienced depression or anxiety in the prior 12 months (Table 1). In the multivariable model with psychological job demands (Table 3), workers handling angry clients were more likely to experience poor mental health: 2.56 times more likely for almost all of the time (adjusted odds ratio, aOR), 2.20 times for 75% of their working time, and 1.74 times for 50% and 25% of their working time. Poor mental health was also more frequent when hiding feelings always (aOR = 3.81) and most of the time (aOR = 2.91) (Table 3). Poor mental health was higher if the respondent was female (aOR = 1.35), had bad general health status (aOR = 14.56), had high psychological demands (aOR = 1.38), rarely or almost never received social support from supervisors (aOR = 1.80), and had job insecurity (aOR = 3.18). The model focusing on job strain generated similar results: the more one handled angry clients and hid feelings, the more likely they were to experience poor mental health. Workers in the passive job group had 1.63 times the risk compared to the low-strain group.

In logistic regression models stratified by gender, the risk of poor mental health with HAC > 50% and high HF was higher for men (aOR = 4.63) than for women (aOR = 3.89) compared to those who never HAC and with low HF (Figure 2). When stratified by social support, the odds ratios were lower for those with high social support from supervisors. Workers with HAC > 50% and high HF were 7.17 times more likely to experience poor mental health when only “sometimes” receiving social support and 13.11 times more when “rarely” or “almost never” (Figure 3).

## 4. Discussion

This large-scale study assessed the prevalence of two key elements of emotionally demanding work in the Korean economy and their associations with depression and anxiety. Handling angry clients and hiding one’s feelings each increased the odds of poor mental health separately; the main effect of HF was slightly greater than that of HAC across the entire range of exposure. When they were combined, there was a roughly additive effect on risk with a nearly five-fold increase in the odds of poor mental health for those with the highest exposures to both emotional demands. Other risk factors were the preventable work exposures of (1) low social support, (2) high psychological demands, (3) low job insecurity, and (4) working >60 h/week. The first three of these also exacerbated the effects of emotional demands (HAC and HF) on the risk of poor mental health.

Our study results are in line with previous studies on high emotional demands, which are defined either as HF alone [6,24] or the combination of HAC and HF [8,9]. However, unlike previous analyses [6,8,9,12,18], we included workers of all employment statuses, from self-employed to independent contractors, who each dealt directly with customers, passengers, pupils, or patients. Fifty-three percent of all those frequently working with clients were in occupational categories outside the “sales” and “service” classifications, which have typically been the focus of previous investigations. These results thus provide a much broader understanding of (negative) emotional demands in previously overlooked occupations such as healthcare, education, social workers, delivery workers, and bus/taxi drivers.

These findings are plausible in light of knowledge about emotional dissonance. Following exposure to a negative stimulus such as handling an angry client, the worker may engage in two types of emotional regulation [25]: attempts to modify or change felt emotions (antecedent-focused) or attempts to modify or suppress expressions (response-focused). The response-focused strategy of hiding one’s feelings is considered an indicator of emotional dissonance between felt and expressed emotions [3]. Emotional dissonance is known for its detrimental psychological effects on mental health [26,27], and the extent to which dissonance exists between felt and expressed emotions affects the degree of harmful consequences [28].

In one prior study, hiding feelings was associated with increased risk of depression or anxiety in both genders [8], while two other studies found this in women only [5,9]. In the present study, female workers showed a slightly higher overall prevalence of depression or anxiety than men, but men had higher vulnerability to the effects of each component of emotional demands (Table 3 and Figure 2). Considering gender discrimination in South Korea, stemming from the hierarchical and patriarchal mindset of Confucian culture [29], the intensity of emotional demands for females is presumed to be severe, thus having more negative effects on mental health. However, our study showed a different result. Additionally, prior studies have examined men and women separately with inconsistent results [5,8,9]. It remains to be understood whether these discrepant findings follow from gender differences in reporting exposures/symptoms or different intensities of exposure among the occupational classifications selected by various investigators.

Social support affects emotional regulation and buffers the detrimental effects of emotional dissonance [3]. In this study, high social support from supervisors showed a particularly large protective effect for mental health, even among those with high exposure to HAC and HF. These results are in line with a previous study [30] and support the suggestion by Aung and Tewogbola [31] that supervisors’ level of authority over working class employees is particularly impactful. In some sectors, such as healthcare, education, and social work, it is not feasible to prevent all negative client encounters; however, workers can be supported in voicing their resulting feelings in a safe space in order to reduce adverse mental health outcomes. As a consequence, it is important to intervene across the full range of psychosocial stressors to protect the mental health of these workers.

Job insecurity has been known to increase the risk of depression [24,32,33]. Among call center workers simultaneously exposed to excessive emotional labor and high job insecurity, the odds of depressive symptoms was 10.13 (95% CI: 3.51–29.23), which is far more than the additive risk [16]. The bivariate association between job insecurity and poor mental health was also present in our study, although the data were too sparse for a stable estimation of effect modification.

High psychological demand (aOR = 1.38) and passive jobs (aOR = 1.63) were shown to increase the risk of poor mental health, as in previous studies [24]. The lack of association between low decision latitude and poor mental health was surprising, as it has been reported in many previous studies [24,34,35]. Another study reported a significant interaction between hiding feelings and job control in relation to depressive mood [5]; job control was measured with only two items. We were able to obtain nine items for decision latitude from the KWCS questionnaire. Although this did not perfectly reproduce the original instrument [22], it generated a fairly comprehensive scale, with three items for decision authority and six items for skill discretion. Most studies on job strain have used the same terminology of psychological demand and decision latitude, although they sometimes measure them with slightly different items. As a result, comparisons between studies may be affected by measurement discrepancies.

In this cross-sectional study, we cannot confirm the direction of any causal relationship. Reverse causation is possible because depressed or anxious workers might over-report emotional demands. If we used only hiding feelings as an exposure variable, as in previous studies [6,18,24], this counterargument would be more difficult to refute. However, it seems less plausible to argue that the frequency of handling angry clients is a consequence of a worker’s poor mental health. While it is reasonable to interpret our findings as emotional demands having detrimental effects on mental health, prospective findings and/or a workplace intervention with a randomized controlled trial would be valuable to confirm causal inference. A related potential weakness is that selection bias could have occurred, such as through the healthy worker effect, in which workers with severe depression would have left or been excluded from the worksite. This could diminish the prevalence of the outcome and could also weaken the strength of association, if it was differential with regard to emotional work demands. Third, the inclusion of “general health” may have artificially reduced the strength of the estimated associations, because mental health is strongly associated with general self–rated health [36]. In this cross-sectional dataset, the onset time of any “general” health problems relative to that of anxiety or depression cannot be determined. A fourth issue is that we had only one statement each for the outcome variable and the two primary risk factors. Each of these may fluctuate within a person over time, so there is possible misclassification. Fifth, the KWCS was not developed to answer causal questions but to monitor working conditions. Nonetheless, previous studies have revealed that items constructed from the KWCS have high external validity, content validity, and reliability [20,22]. Sixth, the data are ten years old. The overall prevalence of occupational risk factors such as low social support, home–work interface, work demands, and job insecurity may have changed, along with emotional demands and mental health outcomes [37]. These adverse exposures and health effects have increased with time and especially during the COVID-19 pandemic [38]. Lastly, we had no data on personal characteristics such as dispositional affectivity and emotional intelligence, which influence emotional regulation [3,39] and play a critical role in the development of depression or anxiety [40].

Nevertheless, this study examined a comprehensive range of psychosocial factors at work and identified important roles for emotional demands as well as social support, job insecurity, and psychological demands. We believe that these findings have important policy implications because of the scope of the analysis and the wide range of occupations included.

## 5. Conclusions

Emotional demands are widespread in the entire modern economy and are more evident in precarious work, which is also becoming far more common. Emotional demands have a marked positive association with poor mental health and also interact with other job stressors. As preventive measures, we recommend to reduce emotional demands, psychological demands, and job insecurity, and increase social support from supervisors to promote mental health.

## Figures and Tables

**Table 1:** Demographic characteristics and psychosocial factors at work among Korean client-facing workers by mental health status.

Variables		No Dep/Anx *n*	Dep/Anx *n*	Dep/Anx %	Total *n*	*p*
Total		23,655	334	1.4	23,989	
Gender	Male	12,623	148	1.2	12,771	0.001
	Female	11,032	186	1.7	11,218	
Age (years old)	20–29	2857	36	1.2	2893	0.210
	30–39	6508	78	1.2	6586	
	40–49	8231	131	1.6	8362	
	50–60	6059	89	1.4	6148	
Education	Middle school or below	1784	31	1.7	1815	0.532
	High school	10,247	151	1.5	10,398	
	2 years in college	4710	59	1.2	4769	
	4 years in university or more	6914	93	1.3	7007	
Income, monthly (million KRW)	<1.3	4383	92	2.1	4475	<0.001
	1.3 to <2.0	6006	83	1.4	6089	
	2.0 to <3.0	7074	76	1.1	7150	
	≥3	6135	82	1.3	6217	
General health status	Good	17,141	125	0.7%	17,266	<0.001
	Fair	6009	143	2.3%	6152	
	Bad	505	66	11.6%	571	
Employment status	Self-employed without employees	6272	93	1.5	6365	0.524
	Self-employed with employees	2715	40	1.5	2755	
	Employees	13,397	178	1.3	13,575	
	Unpaid family workers	771	12	1.5	783	
	Other employees	500	11	2.2	511	
Occupational classification	Managers	443	6	1.3	449	0.546
	Professionals and related workers	4083	56	1.4	4139	
	Clerks	2853	38	1.3	2891	
	Service workers	4332	72	1.6	4404	
	Sales workers	6683	102	1.5	6785	
	Skilled agricultural, forestry and fishery workers	135	0	0.0	135	
	Craft and related trades workers	1934	27	1.4	1961	
	Equipment, machine operating and assembly workers	1802	19	1.0	1821	
	Elementary workers	1379	14	1.0	1393	
	Armed forces	11	0	0.0	11	
Handling angry clients (HAC)	Almost, always	755	33	4.2	788	<0.001
	75%	616	17	2.7	633	
	50%	1211	26	2.1	1237	
	25%	3648	72	1.9	3720	
	Almost, never	17,425	186	1.1	17,611	
Hiding feelings (HF)	Always	1656	52	3.0	1708	<0.001
	Most of the time	6258	118	1.9	6376	
	Sometimes	8635	94	1.1	8729	
	Rarely	5233	57	1.1	5290	
	Never	1873	13	0.7	1886	
Emotional demands (HAC and HF)	HAC > 50% and HF high	958	44	4.4	1002	<0.001
	HAC > 50% and HF low	1624	32	1.9	1656	
	HAC 25% and HF high	1181	43	3.5	1224	
	HAC 25% and HF low	2467	29	1.2	2496	
	HAC never and HF high	5775	83	1.4	5858	
	HAC never and HF low	11,650	103	0.9	11,753	
Psychological demands	Low	11,960	128	1.1	12,088	<0.001
	High	11,691	206	1.7	11,897	
Decision latitude	Low	13,057	190	1.4	13,247	0.538
	High	10,598	144	1.3	10,742	
Job strain	Low strain	7059	73	1.0	7132	<0.001
	High strain	5696	89	1.5	5785	
	Passive group	5995	117	1.9	6112	
	Active group	4901	55	1.1	4956	
Social support from colleagues	High	9949	131	1.3	10,080	0.370
	Intermediate	5150	77	1.5	5227	
	Low	2053	37	1.8	2090	
	Does not apply	6503	89	1.4	6592	
Social support from supervisors	High	7032	69	1.0	7101	<0.001
	Intermediate	4229	58	1.4	4287	
	Low	1958	48	2.4	2006	
	Does not apply	10,436	159	1.5	10,595	
Weekly working hours	35–40	5463	73	1.3	5536	0.017
	41–48	4303	67	1.5	4370	
	49–54	3211	40	1.2	3251	
	55–60	5484	59	1.1	5543	
	61 and over	5194	95	1.8	5289	
Job insecurity	Yes	663	34	4.9	697	<0.001
	No	22,992	300	1.3	23,292	

*p*-value from Pearson chi-squared test statistic; a total of 58 subjects were missing income, 4 subjects—psychological demands, and 4 subjects—job strain.

**Table 2:** Emotional demands by employment status and occupational classification.

		High HF		Low HF		HAC > 50%		HAC 25%		HAC Never	
		*n*	%	*n*	%	*n*	%	*n*	%	*n*	%
Employment status	Self-employed without employees	2212	34.8	4153	65.2	568	8.9	912	14.3	4885	76.7
	Self-employed with employees	1095	39.7	1660	60.3	278	10.1	445	16.2	2032	73.8
	Paid employees	4373	32.2	9202	67.8	1713	12.6	2183	16.1	9679	71.3
	Unpaid family workers	215	27.5	568	72.5	42	5.4	101	12.9	640	81.7
	Informal or other employees	189	37.0	322	63.0	57	11.2	79	15.5	375	73.4
Occupational classification	Managers	135	30.1	314	69.9	43	9.6	70	15.6	336	74.8
	Professionals and related workers	1507	36.4	2632	63.6	421	10.2	488	11.8	3230	78.0
	Clerks	886	30.6	2005	69.4	365	12.6	433	15.0	2093	72.4
	Service workers	1516	34.4	2888	65.6	587	13.3	786	17.8	3031	68.8
	Sales workers	2471	36.4	4314	63.6	679	10.0	1063	15.7	5043	74.3
	Skilled agricultural, forestry and fishery workers	40	29.6	95	70.4	23	17.0	12	8.9	100	74.1
	Craft and related trades workers	578	29.5	1383	70.5	164	8.4	288	14.7	1509	77.0
	Equipment, machine operating and assembly workers	556	30.5	1265	69.5	218	12.0	393	21.6	1210	66.4
	Elementary workers	393	28.2	1000	71.8	156	11.2	186	13.4	1051	75.4
	Armed forces	2	18.2	9	81.8	2	18.2	1	9.1	8	72.7
Total		8084	33.7	15,905	66.3	2658	11.1	3720	15.5	17,611	73.4

**Table 3:** Odds ratios for poor mental health, with multivariable logistic regression models including all variables shown in the table.

		Model with Psychological Demands			Model with Job Strain		
		aOR	95% CI		aOR	95% CI	
Gender	Male	Reference			Reference		
	Female	1.35	1.06	1.72	1.36	1.07	1.73
Income, monthly (million KRW)	<1.3	1.12	0.80	1.57	1.14	0.81	1.60
	1.3 to <2.0	0.90	0.65	1.26	0.91	0.65	1.27
	2.0 to <3.0	0.79	0.57	1.09	0.79	0.57	1.09
	≥3	Reference			Reference		
General health status	Good	Reference			Reference		
	Fair	3.00	2.34	3.84	3.03	2.37	3.88
	Bad	14.56	10.50	20.20	14.61	10.53	20.27
Handling angry clients (HAC)	Almost, always	2.56	1.71	3.83	2.64	1.76	3.95
	75%	2.20	1.31	3.70	2.21	1.32	3.72
	50%	1.74	1.13	2.67	1.75	1.14	2.68
	25%	1.74	1.31	2.31	1.74	1.31	2.30
	Almost, never	Reference			Reference		
Hiding feelings (HF)	Always	3.81	2.04	7.14	3.72	1.99	6.97
	Most of the time	2.91	1.61	5.24	2.86	1.59	5.16
	Sometimes	1.55	0.85	2.81	1.53	0.85	2.78
	Rarely	1.62	0.88	3.01	1.62	0.87	3.00
	Never	Reference			Reference		
Psychological demand	Low	Reference			Reference		
	High	1.38	1.09	1.75			
Job strain	Low strain				Reference		
	High strain				1.17	0.81	1.67
	Passive group				1.63	1.20	2.22
	Active group				1.06	0.73	1.55
Social support from supervisors	Almost always, often	Reference			Reference		
	Sometimes	1.37	0.96	1.96	1.40	0.97	2.00
	Rarely, almost never	1.80	1.21	2.68	1.90	1.28	2.83
	Does not apply	1.51	1.10	2.07	1.42	1.02	1.98
Weekly working hours	35–40	Reference			Reference		
	41–48	1.13	0.80	1.60	1.14	0.81	1.60
	49–54	0.79	0.53	1.18	0.79	0.53	1.18
	55–60	0.72	0.50	1.03	0.72	0.50	1.04
	61 and over	0.98	0.69	1.38	0.97	0.69	1.37
Job insecurity	Yes	3.18	2.14	4.71	3.22	2.17	4.77
	No	Reference			Reference		

## Data Availability

The raw-data of KWCS (Korean Working Conditions Survey) was offered from Occupational Safety and Health Research Institute and available at https://oshri.kosha.or.kr/oshri/researchField/workingEnvironmentSurvey.do (accessed on 23 March 2022).

## Appendix A. Psychosocial Factors at Work

**Table A1:** Psychological demands (4 items).

	High		Low
PD1. Working at very high speed (7 responses)	All of the time	Half	Never
PD2. Working to tight deadlines (7)	All of the time	Half	Never
PD3. Not having enough time to get the job done (5)	Always	Sometimes	Never
PD4. Interrupting a task to take on an unforeseen task (4)	Very often	Occasionally	Never

**Table A2:** Decision latitude (9 items).

	Low		High
SD1. Monotonous tasks (2)	Yes		No
SD2. Learning new things (2)	No		Yes
SD3. Applying own ideas in work (5)	Never	Sometimes	Always
DL1. Working hours determined by oneself (4)	No	Partially *	Yes
DL2. Being able to choose or change your order of tasks (2)	No		Yes
DL3. Being able to choose or change your methods of work (2)	No		Yes
DL4. Being able to choose or change your speed or rate of work (2)	No		Yes
DL5. Influence over the choice of your working partners (5)	Never	Sometimes	Always
DL6. Being able to take a break when one wishes (5)	Never	Sometimes	Always

* determined by oneself under restriction.

**Table A3:** Social support (2 items).

	High	Intermediate	Low	Does Not Apply
SS1. Your colleagues help and support you (6)	Always Most of the time	Sometimes	Rarely Never	No coworker
SS2. Your manager helps and supports you (6)	Always Most of the time	Sometimes	Rarely Never	No supervisor
